# Evaluation of platelet concentrate prescription in pediatric patients at a tertiary care hospital

**DOI:** 10.31744/einstein_journal/2019AO4720

**Published:** 2019-08-14

**Authors:** Camila Augusta Victorino, João Carlos Pina Faria, Fabíola Isabel Suano-Souza, Roseli Oselka Saccardo Sarni

**Affiliations:** 1 Faculdade de Medicina do ABC, Santo André, SP, Brazil; 2 Universidade Federal de São Paulo, São Paulo, SP, Brazil

**Keywords:** Transfusion medicine, Platelet transfusion, Child, Emergency treatment, Hemorrhage

## Abstract

**Objective:**

To verify the adequacy of platelet concentrate prescription by pediatricians in different pediatric sectors of a general hospital.

**Methods:**

A cross-sectional study evaluating 218/227 platelet concentrate records in children and adolescents (zero to 13 years old), from January 2007 to April 2015, by the pediatricians of the emergency room, sick bay and intensive care unit. The requisitions were excluded in patients with hematological diseases and those without the number of platelets.

**Results:**

Children under 12 months received 98 platelet concentrates (45.2%). Most of the transfusions were prophylactic (165; 79%). Regarding the transfusion site, 39 (18%) were in the emergency room, 27 (12.4%) in the sick bay and 151 (69.6%) in the intensive care unit. The trigger, prescribed volume and platelet concentrate subtype were adequate in 59 (28.2%), 116 (53.5%) and 209 (96.3%) of the transfusions, respectively. Patients with hemorrhage presented adequacy in 42 (95.5%), while children without bleeding presented in 17 (10.3%). The most common inadequacy related to volume was the prescription above recommendation (95; 43.8%). Eight platelet concentrates were prescribed with subtype requests without indication.

**Conclusion:**

The results obtained in this study showed that transfusion of platelet concentrate occurred more adequately in children with active bleeding compared to prophylactic transfusion. There was a tendency to prescribe high volumes and platelet subtypes not justified according to current protocols. The teaching of transfusion medicine should be more valued at undergraduate and medical residency.

## INTRODUCTION

The first successful human blood transfusion in history was performed by a British obstetrician, James Blundell, in 1818.^(^^1^^)^ In the 1950´s, it was possible to fractionate platelets from whole blood.^(^^2^^)^ In 1961, the impact of platelet transfusion on mortality reduction of oncological patients was confirmed,^(^^3^^)^ and its started to be prescribed more frequently.^(^^4^^)^

Despite being the second most transfused blood component after packed red blood cells (PRBC), platelet concentrate (PC) transfusion is the major cause of transfusion reaction in patients with multiple transfusions;^(^^5^^)^ in that, the most frequent events are febrile non-hemolytic reaction, allergic reaction, and bacterial sepsis.^(^^6^^)^ Contrary to the transfusion of PRBC, in which one unit at a time is prescribed in most situations, in platelet transfusions several units are given (one unit of PC for every 10kg/weight), exposing the recipient to more donors, and consequently, to a higher risk of transfusion reactions.^(^^7^^)^ Additionally, platelet storage at 22°C increases the risk of bacterial contamination of this blood component.^(^^4^^)^

The most frequent indications for platelet transfusion are low platelet count, hemorrhage, and use of anticoagulants.^(^^8^^)^

The management guides for PC transfusion in Pediatrics are based on those for adults,^(^^9^^)^ and recommend the prophylactic transfusion of platelets only when the platelet count is below 10,000/mm^3^, below 20,000/mm^3^ in patients with a risk factor such as sepsis, below 50,000/mm^3^ in a non-critical surgery or invasive procedure, and below 100,000/mm^3^ in cranial and ocular surgeries.^(^^10^^)^ In cases of hemorrhage, transfusion is indicated when the platelet count is below 50,000/mm^3^ in stable children, below 100,000/mm^3^ in children with critical conditions, and at any value in case of patients with a qualitative defect of the platelets.^(^^11^^)^

The appropriate order for the PC subtype (filtered, irradiated, and washed) influences the result of transfusion, and reduces the risk of transfusion reactions.^(^^7^^,^^12^^)^ Filtered PC eliminates 99% of leukocytes and is indicated primarily to avoid the febrile non-hemolytic reaction in patients with multiple transfusions, and the transmission of cytomegalovirus in immunosuppressed individuals.^(^^7^^)^ Irradiated PC prevents the clonal proliferation of donor lymphocytes in immunosuppressed recipients, avoiding the graft *versus* host disease.^(^^7^^)^ Washed PC has the largest quantity possible of its plasma replaced by 0.9% saline solution, with the objective of removing the plasma proteins that might cause anaphylactic reactions in individuals with a congenital deficiency of a serum protein, or who have already presented with a severe allergic reaction to a previous transfusion.^(^^7^^)^

Platelet concentrate transfusion out of the current recommendations elevates the risk for complications and death. The prescription of low volumes results in a lower output than ideal, and high volumes are associated with the risk of circulatory overload. The choice of incorrect procedures increases the incidence of preventable transfusion reactions, besides increasing the costs of treatment.^(^^12^^)^

A study carried out in Brazil, in 2016, evaluated the knowledge of sixth-year medical undergraduate students and residents from several areas. Transfusions were adequate in 35% and 49.5%, respectively, demonstrating little knowledge on the subject.^(^^13^^)^

Platelet concentrate transfusion is an important and frequent strategy in various clinical situations. Even so, there are few studies available describing and assessing this procedure for the pediatric age range.^(^^14^^–^^17^^)^

## OBJECTIVE

To evaluate the adequacy of the prescription of platelet concentrate by pediatricians, taking into consideration the transfusion trigger, the prescribed volume, and the order for platelet subtypes, in three sectors of the hospital, namely emergency department, ward, and intensive care unit.

## METHODS

This was a cross-sectional study based on the survey of requisition records for transfusion procedures in children and adolescents aged from zero to 13 years, at the *Hospital e Pronto-Socorro Central* (HPSC), in the city of São Bernardo do Campo (SP), Brazil. The records were completed by the pediatricians of the three hospital sectors: emergency department, ward, and intensive care unit (ICU) during the period from January 2007 to April 2015.

Throughout the study period, Pediatrics was composed of 4 beds in the emergency room, 5 observation beds, 40 ward beds, and five in the pediatric ICU. The hospital did not have an Obstetrics Center, Nursery, Operating Room, or an Oncology/Hematology service, but relied on the transfusion agency (COLSAN – *Associação Beneficente de Coleta de Sangue*) that stored some blood components and performed simple tests, such as direct and reverse ABO blood typing, Rh typing, and cross-testing. Whenever a subtype of blood component requested was not available at the transfusion agency, this was sent by the main office, which is in the city of São Paulo (SP), Brazil. The agency had PC obtained from whole blood, but rarely from aphaeresis.

Inclusion criteria were PC transfusions performed in children seen in the emergency department, ward, and pediatric ICU during the study period. Excluded transfusions were those that had an incomplete record, hindering analysis of transfusion adequacy.

To evaluate the adequacy of the transfusion trigger, the volume prescribed and the choice of PC subtypes, we used the recommendations of the Ministry of Health, of 2015.^(^^7^^)^ During the study period, no unit obtained from apheresis was transfused.

The data collected from the requisition forms were general, such as age (months), sex, present disease, and location of the transfusion request (emergency room, ward, or pediatric ICU); and data relative to the characteristics of the transfusion, including trigger (reason for transfusion), prescribed volume, and PC subtype. As to the trigger, assessment was made of whether the transfusion was prophylactic (based only on platelet count) or performed in patients with active hemorrhage. Prophylactic PC transfusions were considered adequate in patients with less than 10,000 platelets/mm^3^. Transfusions with higher counts were evaluated individually, taking into consideration the presence of associated morbidities and the clinical status. As to the volume prescribed, a volume between 5 and 10mL/kg in infants under one year, and one unit of PC for every 10kg in older children were considered adequate. In PC subtypes, the requisition of filtered, irradiated, and washed components was evaluated, taking into account the patient's present disease and transfusion history.

The study was approved by the Research Ethics Committee of the *Faculdade de Medicina do ABC* (opinion No. 2.001.832), CAAE: 62730716.1.0000.0082.

Data were entered and consolidated on an Excel (Microsoft) spreadsheet, having been analyzed with the Statistical Package for Social Science (SPSS), version 24.0. The qualitative variables were presented as absolute number and percentage; continuous variables were analyzed as to their distribution, presented as mean±standard deviation when parametric, and median (minimum and maximum) when non-parametric. For comparison of the qualitative data, we used the χ^2^. The significance level adopted was 5%.

## RESULTS

**Figure 1:**
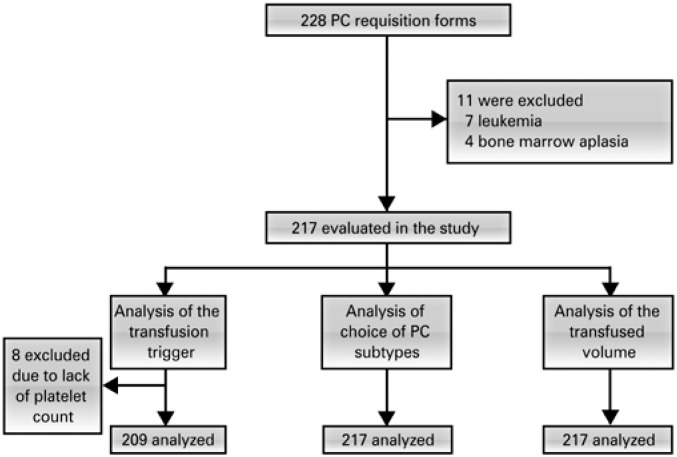
Study flowchart PC: platelet concentrate.

The cases included 217 of the 228 PC transfusion requisitions made during the study period. Eleven requisitions were excluded of patients with hematological disease (7 cases of leukemia and 4 of bone marrow aplasia), since the patients were transferred with the ongoing management of an external hematologist, or the recorded data was not clear. Also excluded were eight specific requisitions for analysis of the transfusion trigger due to lack of registration of the platelet count, although these requisitions were used for the analyses of prescribed volume and subtypes of platelets (Figure 1).

In the sample studied, PC transfusions in the male sex predominated (140; 64.5%). The mean and median ages of patients who received PC were, respectively, 47.4±52.8 months and 17 months (1 to 156 months); 98 (45.2%) transfusions were performed on children aged less than 12 months.

There were descriptions of the presence of disease in 217 requisitions, 109 (50.2%) of which reported sepsis (Table 1). As to the location of the transfusion, 39 (18%) were done in the emergency department, 27 (12.4%) in the ward, and 151 (69.6%) in the pediatric ICU (Table 1).

**Table 1:** General characteristics of platelet transfusions

Variable		n (%)
Sex, n=217		
	Male	140 (64.5)
	Female	77 (35.5)
Age, years, n=217		
	<1	98 (45.2)
	1<5	41 (45.2)
	5-13	78 (35.9)
Existing disease, n=217		
	Sepsis	109 (50.2)
	Idiopathic thrombocytopenic purpura	12 (5.5)
	Hemolytic uremic syndrome	9 (4.1)
	Heart disease	9 (4.1)
	Liver disease	5 (2.3)
	Others	15 (6.9)
	Not reported in the requisition	58 (26.7)
Location, n=217		
	Emergency department	39 (18)
	Ward	27 (12.4)
	Pediatric ICU	151 (69.6)
Presence of hemorrhage, n=209*		
	Yes	44 (21)
	No	165 (79)
Platelet count, n=209*		
	<10,000/mm^3^	24 (11.5)
	10,000/mm^3^<30,000/mm^3^	73 (34.9)
	30,000/mm^3^<50,000/mm^3^	76 (36.4)
	50,000/mm^3^<100,000/mm^3^	29 (13.9)
	≥100,000/mm^3^	7 (3.3)
PC prescribed volume (recommendation), n=217		
	Lower	6 (2.8)
	Adequate	116 (53.4)
	Higher	95 (43.8)
PC subtype, n=217		
	Simple	209 (96.3)
	With requested subtype†	8 (3.7)
	Filtered	7
	Irradiated	5
	Washed	1

* The platelet concentrate requisitions with no record of platelet count were excluded;

† in some platelet concentrate requisitions, more than one subtype was ordered, therefore the sum of filtered, irradiated and washed is greater than the number of platelet concentrate with requested subtype. ICU: intensive care unit; PC: platelet concentrate.

Most of the transfusions (165; 79%) were performed on children who did not present with active bleeding, and were based only on platelet count in the complete blood count (CBC). The platelet count was lower than 50,000/mm^3^ and 10,000/mm^3^ in 173 (82.8%) and 24 (11.5%) of the transfusions performed, respectively.

The trigger, prescribed volume, and the subtype of PC were appropriate in 59 (28.2%); 116 (53.4%), and 209 (96.3%) transfusions, respectively (Table 2). There were requisitions for one or more subtypes of PC in eight transfusions (3.7%). The most requested subtype was filtered, in seven requisitions. The most common inadequacy relative to volume was the prescription for more than what is recommended (95; 43.8%). Transfusion adequacy was greatest in situations in which there was active hemorrhage in comparison with prophylactic transfusions (42; 95.5% *versus* 17; 10.3%; p<0.001).

**Table 2:** Description of the variables assessed regarding adequacy of platelet concentrate transfusion

Variable		n (%)
Presence of hemorrhage, n=209*		
	Yes	44 (95.5)
	No	165 (10.3)
Adequate trigger, n=209*		59 (28.2)
	PC prescribed volume (recommendation), n=217	
	Lower	6 (0)
	Adequate	116 (100)
	Higher	95 (0)
PC subtype, n=217		
	Simple	209 (100)
	With requested subtype†	8 (0)
	Filtered	7
	Irradiated	5
	Washed	1

* The platelet concentrate requisitions with no record of platelet count were excluded;

† in some platelet concentrate requisitions, more than one subtype was ordered, therefore the sum of filtered, irradiated and washed is greater than the number of platelet concentrate with requested subtype. PC: platelet concentrate.

Median platelet count between the groups with adequate PC transfusions was lower than that in which the transfusions were considered inadequate (18,000/mm³; 2,000-50,000/mm³ *versus* 32,500/mm³; 5,000-166,000/mm³; p<0.001).

When the location of the transfusion was evaluated as to the adequacy of the trigger, volume, and subtype, it was noted that the emergency department had the highest trigger adequacy in 40.5% (p=0.137), albeit without a statistically significant difference among the sectors, the ward relative to volume (p=0.032), and the ICU as to subtype (p=0.001) (Table 3).

**Table 3:** Adequacy of trigger, volume and subtype in three hospital sectors

Adequacy	Emergency department	Ward	ICU	p value*
Trigger, n=209	15/37 (40.5)	5/26 (19.2)	39/146 (26.7)	0.137
Volume, n=217	25/39 (64.1)	19/27 (70.4)	72/151 (47.7)	0.032
Subtype, n=217	36/39 (92.3)	23/27 (85.2)	150/151 (99.3)	0.001

* Significance level of the χ^2^ test. Results expressed as n/n total (%).

ICU: intensive care unit.

## DISCUSSION

The study demonstrated that there was adequacy of the transfusion trigger in only 28.2% of the PC requisitions. The prescribed volume and the requests for subtypes of PC were adequate in 53.5% and 96.3%, respectively.

As to the transfusion trigger, adequacy was superior in children with active hemorrhage (95.5%) when compared to prophylactic transfusions (10.3%). Four retrospective studies carried out at tertiary care hospitals, and based on the recommendations of the American Association of Blood Banks, similar to the indications of the Brazilian Ministry of Health, found superior adequacies: a Venezuelan study with 404 children verified a 52.6%^(^^14^^)^ adequacy; a Canadian registry verified 139 PC transfusions and showed adequacy in 64.7%,^(^^15^^)^ a rate close to that of an Indian study, which evaluated 566 PC transfusions and found 66.7% adequacy.^(^^16^^)^ On the other hand, a Malaysian study with 119 PC transfusions presented with 81.5% adequacy.^(^^17^^)^

When there was inadequacy in the calculation of the prescribed volume, the predominant error was excessive volume, representing 43.8% of total, increasing the risk of transfusion-related circulatory overload, a transfusion reaction that presents with 12% mortality.^(^^12^^)^

Despite the high level of adequacy of requisition of simple PC (with no subtype), all 13 subtypes requested in 8 transfusions were incorrect. When not available in storage, the preparation of a PC subtype requires a waiting period for receipt of the blood component, which can be decisive for a severely ill patient. We found no studies as to volume adequacy and choice of PC subtypes.

Among the hypotheses raised to justify the results found are lack of teaching of this topic in medical undergraduate course and medical residency, lack of knowledge of pediatricians who treat severely-ill children about PC transfusion protocols, and lack of a continuing education program on blood transfusion.

Transfusion inadequacy raises the incidence of transfusion reactions and death. On the other hand, it can reduce the stocks of PC, which is the blood component with shorter shelf life (5 days) and increase the costs of treatment.^(^^18^^)^

The data from this study were forwarded to the Transfusion Committee of the hospital, with the proposal of conducting a refresher course for the pediatric team. The existence of a Transfusion Committee is mandatory in every organization performing transfusions of blood components (ordinance 158/GM/MS, dated February 4^th^, 2016).^(^^19^^)^ The Transfusion Committee is responsible for monitoring the practice blood transfusion at the healthcare organization, continuing education activity in blood transfusion, in addition to hemovigilance and preparation of protocols for routine blood transfusion.^(^^19^^)^

As to the limitations, we can describe the fact it is a retrospective study dependent on information recorded by different physicians, who often do not properly fill in the transfusion requisition forms.^(^^7^^)^

## CONCLUSION

Adequacy in the indication of platelet transfusion was very low when compared to other records. Adequacy in prescription of platelet concentrate was greater in children with active hemorrhage. There was an inclination towards requests for excessive volumes and platelet subtypes, which were not justified as per the current transfusion protocols. These data reinforce the need for teaching of transfusion medicine to be given more value in medical undergraduate course and residency. Moreover, the hospital, along with the Transfusion Committee, should provide continuing education on the topic when the need is identified.
