# Supplementary material for: Evaluation of platelet concentrate prescription in pediatric patients at a tertiary care hospital
Source: Einstein (Sao Paulo). 2019 Aug 14;17(4):eAO4720. doi: 10.31744/einstein_journal/2019AO4720 (PMC6706223; doi:10.31744/einstein_journal/2019AO4720)
Supplement: Translation

# Translation: Avaliação da prescrição de concentrado de plaquetas em pacientes pediátricos em hospital terciário

**Authors:** Camila Augusta Victorino, João Carlos Pina Faria, Fabíola Isabel Suano-Souza, Roseli Oselka Saccardo Sarni

**Affiliations:** 1 Faculdade de Medicina do ABC, Santo André, SP, Brasil; 2 Universidade Federal de São Paulo, São Paulo, SP, Brasil

**Keywords:** Medicina transfusional, Transfusão de plaquetas, Criança, Tratamento de emergência, Hemorragia

## Abstract

**Objetivo:**

Verificar a adequação na prescrição de concentrado de plaquetas por pediatras em diferentes setores da pediatria de um hospital geral.

**Métodos:**

Estudo transversal avaliando 218/227 fichas de requisição de concentrado de plaquetas de crianças e adolescentes (zero a 13 anos), de janeiro de 2007 a abril de 2015 pelos pediatras do pronto-socorro, enfermaria e unidade de terapia intensiva. Excluíram-se as requisições em portadores de doenças hematológicas e aquelas sem o número de plaquetas.

**Resultados:**

Crianças com menos de 12 meses receberam 98 prescrições de concentrado de plaquetas (45,2%). A maioria das transfusões foi profiláticas (165; 79%). Em relação ao local da transfusão, 39 (18%) foram no pronto-socorro, 27 (12,4%) na enfermaria e 151 (69,6%) na unidade de terapia intensiva. O gatilho, o volume prescrito e o subtipo de concentrado de plaquetas foram adequados em 59 (28,2%), 116 (53,5%) e 209 (96,3%) das transfusões, respectivamente. Prescrições para pacientes com hemorragia apresentaram adequação em 42 (95,5%) transfusões, enquanto para crianças sem hemorragia houve adequação em 17 (10,3%) vezes. A inadequação mais comum em relação ao volume foi a prescrição acima da recomendação (95; 43,8%). Foram prescritos oito concentrados de plaquetas sem indicação de solicitação de subtipos.

**Conclusão:**

Os resultados obtidos nesse estudo mostraram que a prescrição de transfusão de concentrado de plaquetas foi mais adequada em crianças com hemorragia ativa em comparação com a transfusão profilática. Houve tendência à prescrição de volumes elevados e de subtipos de plaquetas, o que não se justifica segundo os protocolos atuais. O ensino da medicina transfusional deve ser mais valorizado na graduação e na residência médica.

## INTRODUÇÃO

A primeira transfusão de sangue humano bem-sucedida na história foi realizada por um médico obstetra britânico, James Blundell, em 1818.^(^^1^^)^ Na década de 1950, foi possível fracionar as plaquetas do sangue total.^(^^2^^)^ Em 1961, foi comprovado o impacto da transfusão de plaquetas na redução da mortalidade de pacientes oncológicos^(^^3^^)^ e, então, sua prescrição passa a ocorrer com maior frequência.^(^^4^^)^

Apesar de ser o segundo hemocomponente mais transfundido, após o concentrado de hemácias (CH), a transfusão de concentrado de plaquetas (CP) é a maior causa de reações transfusionais em politransfundidos,^(^^5^^)^ sendo as mais frequentes a reação febril não hemolítica, a reação alérgica e a sepse bacteriana.^(^^6^^)^ Contrariamente à transfusão de CH, na qual prescreve-se uma unidade de cada vez na maioria das situações, na transfusão de plaquetas são administradas mais unidades (uma unidade de CP a cada 10kg/peso), expondo o receptor a mais doadores e, consequentemente, a um risco elevado de reações transfusionais.^(^^7^^)^ Além disso, a forma de armazenamento das plaquetas a 22°C aumenta o risco de contaminação bacteriana desse hemocomponente.^(^^4^^)^

As indicações mais frequentes de transfusão de plaquetas são baixa contagem plaquetária, hemorragia e uso de anticoagulantes.^(^^8^^)^

Os guias de conduta de transfusão de CP em pediatria baseiam-se nos de adultos,^(^^9^^)^ e recomendam a transfusão profilática de plaquetas somente quando a contagem plaquetária está abaixo de 10.000/mm^3^, abaixo de 20.000/mm^3^ em pacientes com fator de risco como sepse, abaixo de 50.000/mm^3^ em cirurgia não crítica ou procedimento invasivo, e abaixo de 100.000/mm^3^ em cirurgias cranianas e oculares.^(^^10^^)^ Já em casos de hemorragia, a transfusão está indicada quando a contagem de plaquetas é inferior 50.000/mm^3^ em crianças estáveis, abaixo de 100.000/mm^3^ em crianças graves e em qualquer valor no caso de pacientes portadores de doenças com defeito qualitativo das plaquetas.^(^^11^^)^

A solicitação adequada do subtipo de CP (filtrado, irradiado e lavado) influencia no resultado da transfusão e reduz o risco de reações transfusionais.^(^^7^^,^^12^^)^ O CP filtrado elimina 99% dos leucócitos e é indicado principalmente para evitar a reação febril não hemolítica em politransfundidos e a transmissão de citomegalovírus em imunodeprimidos.^(^^7^^)^ O CP irradiado previne a proliferação clonal de linfócitos do doador no receptor em indivíduos imunodeprimidos, evitando a doença do enxerto contra hospedeiro transfusional.^(^^7^^)^ O CP lavado tem a maior quantidade possível de seu plasma substituída por solução salina 0,9%, com o objetivo de retirar as proteínas plasmáticas que podem causar reação anafilática em indivíduos com deficiência congênita de alguma proteína sérica, ou que já apresentaram reação alérgica grave à transfusão anterior.^(^^7^^)^

A transfusão de CP fora das recomendações atuais eleva o risco de complicações e de morte. A prescrição de baixo volume resulta em rendimento inferior ao ideal, e o alto volume associa-se com risco de sobrecarga circulatória. A escolha de procedimentos incorretos, aumenta a incidência de reações transfusionais preveníveis, além de elevar os custos do tratamento.^(^^12^^)^

Estudo realizado no Brasil em 2016 avaliou o conhecimento de alunos do sexto ano do curso de medicina e residentes de diversas áreas. A adequação transfusional foi de 35% e 49,5% respectivamente, demonstrando pouco conhecimento sobre o tema.^(^^13^^)^

A transfusão de CP é estratégia importante e frequente em várias situações clínicas. Apesar disso, há poucos estudos disponíveis que descrevam e avaliem esse procedimento na faixa etária pediátrica.^(^^14^^–^^17^^)^

## OBJETIVO

Avaliar a adequação da prescrição de concentrado de plaquetas por pediatras, levando-se em conta o gatilho transfusional, o volume prescrito e a solicitação de subtipos de plaquetas e nos três setores do hospital: pronto-socorro, enfermaria e unidade de terapia intensiva.

## MÉTODOS

Foi realizado estudo transversal e baseado no levantamento de fichas de requisição de procedimento hemoterápico de crianças e adolescentes de zero a 13 anos no Hospital e Pronto-Socorro Central (HPSC), em São Bernardo do Campo (SP). As fichas foram preenchidas pelos médicos pediatras dos três setores do hospital: pronto-socorro, enfermaria e unidade de terapia intensiva (UTI) pediátrica no período de janeiro de 2007 a abril de 2015.

No período do estudo, a pediatria era composta por 4 leitos na sala de emergência, 5 de observação, 40 de enfermaria e cinco de UTI pediátrica. O hospital não possuía centro obstétrico, berçário, centro cirúrgico e nem serviço de oncologia/hematologia. Contava com agência transfusional (COLSAN – Associação Beneficente de Coleta de Sangue) que armazenava alguns hemocomponentes e realizava testes simples, como tipagem sanguínea ABO direta e reversa, tipagem Rh e provas cruzadas. Quando era solicitado algum subtipo de hemocomponente não disponível na agência transfusional, este era enviado pela sede, que fica no município de São Paulo (SP). A agência dispunha de CP obtidos de doação de sangue total, mas raramente de aférese.

Os critérios de inclusão foram as transfusões de CP realizadas em crianças atendidas no pronto-socorro, na enfermaria e na UTI pediátrica no período do estudo. Foram excluídas transfusões que possuíam registro incompleto, impossibilitando análise da adequação transfusional.

Para avaliar a adequação do gatilho transfusional, o volume prescrito e a escolha dos subtipos de CP, utilizamos as recomendações do Ministério da Saúde de 2015.^(^^7^^)^ Durante o período do estudo, não foi transfundida unidade obtida por aférese.

Os dados coletados das fichas de requisição transfusional foram gerais, como idade (meses), sexo, doença presente e local da solicitação da transfusão (pronto-socorro, enfermaria ou UTI pediátrica); e dados referentes às características das transfusões como o gatilho (motivo da transfusão), volume prescrito e subtipo de CP. No gatilho, foi avaliado se a transfusão foi profilática (baseado somente no valor das plaquetas) ou realizada em pacientes com hemorragia ativa. Foram consideradas adequadas as transfusões profiláticas de CP em pacientes com menos de 10.000 plaquetas/mm^3^. As transfusões com contagens maiores foram avaliadas individualmente, levando-se em conta a presença de morbidades associadas e o quadro clínico. Em relação ao volume prescrito, considerou-se adequado o volume entre 5 e 10mL/kg em menores de 1 ano e uma unidade de CP a cada 10kg nos maiores. Nos subtipos de CP, avaliou-se a solicitação de componentes filtrados, irradiados e lavados, levando-se em consideração a doença presente e o histórico transfusional do paciente.

O estudo foi aprovado pelo Comitê de Ética em Pesquisa da Faculdade de Medicina do ABC (parecer n° 2.001.832), CAAE: 62730716.1.0000.0082.

Os dados foram digitados e consolidados em planilha Excel (Microsoft), tendo sido analisados no *Statistical Package for Social Science* (SPSS), versão 24.0. As variáveis qualitativas foram apresentadas na forma de número absoluto e percentual; as contínuas foram analisadas quanto à sua distribuição, apresentadas na forma de média±desvio padrão, quando paramétricas, e mediana (mínimo e máximo), quando não paramétricas. Para comparação dos dados qualitativos, utilizamos o teste de χ^2^. O nível de significância adotado foi de 5%.

## RESULTADOS

Incluíram-se, na casuística, 217 das 228 requisições de transfusões de CP solicitadas no período do estudo. Excluíram-se 11 requisições de pacientes com doença hematológica (7 leucemias e 4 aplasias de medula óssea), pois os pacientes foram transferidos já com conduta de hematologista externo ou os dados registrados não estavam claros. Excluímos também oito requisições especificamente da análise do gatilho transfusional devido à falta do registro do valor das plaquetas, porém essas requisições foram utilizadas para as análises de volume prescrito e de subtipos de plaquetas (Figura 1).

**Figura 1:** Fluxograma do estudo CP: concentrado de plaquetas.

Na amostra estudada, predominaram as transfusões de CP no sexo masculino (140; 64,5%). A média e a mediana de idade dos pacientes que receberam CP foram, respectivamente, 47,4±52,8 meses e 17 meses (1 a 156 meses), respectivamente; 98 (45,2%) transfusões foram realizadas em crianças com idade inferior a 12 meses.

Houve descrição de doença presente em 217 requisições, das quais 109 (50,2%) relatavam sepse (Tabela 1). Em relação ao local da transfusão, 39 (18%) foram feitas no pronto-socorro, 27 (12,4%) na enfermaria e 151 (69,6%) na UTI pediátrica (Tabela 1).

**Tabela 1:** Características gerais das transfusões de plaquetas

Variável		n (%)
Sexo, n=217		
	Masculino	140 (64,5)
	Feminino	77 (35,5)
Idade, anos, n=217		
	<1	98 (45,2)
	1<5	41 (45,2)
	5-13	78 (35,9)
Doença presente, n=217		
	Sepse	109 (50,2)
	Púrpura trombocitopênica idiopática	12 (5,5)
	Síndrome hemolítico-urêmica	9 (4,1)
	Cardiopatia	9 (4,1)
	Hepatopatia	5 (2,3)
	Outros	15 (6,9)
	Não relatado da requisição	58 (26,7)
Local, n=217		
	Pronto-socorro	39 (18)
	Enfermaria	27 (12,4)
	UTI pediátrica	151 (69,6)
Presença de hemorragia, n=209*		
	Sim	44 (21)
	Não	165 (79)
Contagem de plaquetas, n=209*		
	<10.000/mm^3^	24 (11,5)
	10.000/mm^3^ <30.000/mm^3^	73 (34,9)
	30.000/mm^3^ <50.000/mm^3^	76 (36,4)
	50.000/mm^3^ <100.000/mm^3^	29 (13,9)
	≥100.000/mm^3^	7 (3,3)
Volume prescrito de CP (recomendação), n=217		
	Menor	6 (2,8)
	Adequado	116 (53,4)
	Maior	95 (43,8)
Subtipo CP, n=217		
	Simples	209 (96,3)
	Com subtipo solicitado†	8 (3,7)
	Filtrada	7
	Irradiada	5
	Lavada	1

* Foram excluídas as requisições de concentrado de plaquetas sem o registro do valor da contagem plaquetária;

† em algumas requisições de concentrado de plaquetas, houve solicitação de mais de um subtipo, por isso a somatória de filtrados, irradiados e lavados é maior que o número de concentrado de plaquetas com subtipo solicitado. UTI: unidade de terapia intensiva; CP: concentrado de plaquetas.

A maioria das transfusões (165; 79%) foi realizada em crianças que não apresentavam sangramento ativo, baseando-se somente no valor das plaquetas do hemograma. O valor das plaquetas estava inferior a 50.000/mm^3^ e 10.000/mm^3^ em 173 (82,8%) e 24 (11,5%) das transfusões realizadas, respectivamente.

O gatilho, o volume prescrito e o subtipo de CP foram adequados em 59 (28,2%); 116 (53,4%) e 209 (96,3%) transfusões, respectivamente (Tabela 2). Houve solicitação de um ou mais subtipos de CP em oito transfusões (3,7%). O subtipo mais solicitado foi o filtrado, em sete requisições. A inadequação mais comum, em relação ao volume, foi a prescrição acima da recomendação (95; 43,8%).

**Tabela 2:** Descrição das variáveis avaliadas em relação à adequação da transfusão de concentrados de plaquetas

Variável		n (%)
Presença de hemorragia, n=209*		
	Sim	44 (95,5)
	Não	165 (10,3)
	Gatilho adequado, n=209*	59 (28,2)
Volume prescrito de CP (recomendação), n=217		
	Menor	6 (0)
	Adequado	116 (100)
	Maior	95 (0)
Subtipo de CP, n=217		
	Simples	209 (100)
	Com subtipo solicitado†	8 (0)
	Filtrada	7
	Irradiada	5
	Lavada	1

* Foram excluídas as requisições de concentrado de plaquetas sem o registro do valor da contagem plaquetária;

† em algumas requisições de concentrado de plaquetas, houve solicitação de mais de um subtipo, por isso a somatória de filtrados, irradiados e lavados é maior que o número de concentrado de plaquetas com subtipo solicitado. CP: concentrado de plaquetas.

A adequação transfusional foi maior nas situações em que ocorreram hemorragia ativa em comparação com transfusões profiláticas (42; 95,5% *versus* 17; 10,3%; p<0,001).

A mediana da contagem de plaquetas entre o grupo cuja transfusão de CP estava adequada foi menor em relação àquele em que a transfusão foi considerada inadequada (18.000/mm³; 2.000-50.000/mm³ *versus* 32.500/mm³; 5.000-166.000/mm³; p<0,001).

Quando se avaliou o local de transfusão em relação à adequação do gatilho, do volume e do subtipo, observou-se que o pronto-socorro teve maior acerto do gatilho em 40,5% (p=0,137), mas sem diferença estatisticamente significativa entre os setores, a enfermaria em relação ao volume (p=0,032), e a UTI quanto ao subtipo (p=0,001) (Tabela 3).

**Tabela 3:** Adequação do gatilho, volume e subtipo nos três setores hospitalares

Adequação	Pronto-socorro	Enfermaria	UTI	Valor de p*
Gatilho, n=209	15/37 (40,5)	5/26 (19,2)	39/146 (26,7)	0,137
Volume, n=217	25/39 (64,1)	19/27 (70,4)	72/151 (47,7)	0,032
Subtipo, n=217	36/39 (92,3)	23/27 (85,2)	150/151 (99,3)	0,001

* Nível de significância do teste do χ^2^. Resultados expressos como n/n total (%).

UTI: unidade de terapia intensiva.

## DISCUSSÃO

O estudo demonstrou que houve adequação do gatilho transfusional somente em 28,2% das solicitações de CP. O volume prescrito e a solicitação de subtipos de CP foram adequados em 53,5% e 96,3%, respectivamente.

Em relação ao gatilho transfusional, a adequação foi superior em crianças com hemorragia ativa (95,5%) quando comparada às transfusões profiláticas (10,3%). Quatro estudos retrospectivos realizados em hospitais terciários baseados nas recomendações da *American Association of Blood Banks*, semelhantes às indicações do Ministério da Saúde, encontraram adequações superiores: um estudo venezuelano com 404 crianças verificou adequação de transfusão de CP de 52,6%;^(^^14^^)^ já registro canadense, que verificou 139 transfusões de CP, apresentou adequação de 64,7%,^(^^15^^)^ taxa próxima à de pesquisa indiana, que avaliou 566 transfusões de CP e encontrou 66,7% de adequação;^(^^16^^)^ já estudo malaio, com 119 transfusões de CP, apresentou 81,5% de adequação.^(^^17^^)^

Quando houve inadequação no cálculo do volume prescrito, o erro que predominou foi o volume excessivo, representando 43,8% do total, aumentando o risco de sobrecarga circulatória relacionada à transfusão, reação transfusional que apresenta 12% de mortalidade.^(^^12^^)^

Apesar da adequação da solicitação de CP simples (sem subtipo) ter sido alta, todos os 13 subtipos solicitados em 8 transfusões estavam incorretos. Quando não está disponível no estoque, o preparo de um subtipo de CP requer maior tempo de espera para o recebimento do hemocomponente, o que pode ser decisivo a um paciente grave. Não encontramos estudos sobre adequação de volume e escolha de subtipos de CP.

Entre as hipóteses levantadas para justificar os resultados encontrados estão a falta do ensino desse tema na graduação e na residência médica, o desconhecimento de pediatras que atuam com crianças graves dos protocolos de transfusão de CP e a falta de programa de educação continuada sobre hemoterapia.

A inadequação transfusional eleva a incidência de reações transfusionais e de morte. Por outro lado, pode reduzir os estoques de CP, que é o hemocomponente de menor validade (5 dias) e aumentar os custos do tratamento.^(^^18^^)^

Os dados deste estudo foram encaminhados ao Comitê Transfusional do hospital, com a proposta de realizar atualização da equipe pediátrica. A existência de um Comitê Transfusional é obrigatória em toda instituição que realiza transfusão de hemocomponentes (portaria 158/GM/MS, de 4 de fevereiro de 2016).^(^^19^^)^ São competências do Comitê Transfusional o monitoramento da prática hemoterápica na instituição de assistência à saúde, a atividade educacional continuada em hemoterapia, e a hemovigilância e a elaboração de protocolos de atendimento da rotina hemoterápica.^(^^19^^)^

Quanto às limitações, podemos descrever o fato de ter sido um estudo retrospectivo dependente de informações registradas por diferentes médicos que, por vezes, não preenchiam adequadamente as fichas de requisição transfusional.^(^^7^^)^

## CONCLUSÃO

A adequação na indicação da transfusão de plaquetas foi muito abaixo quando comparada outros registros. A adequação na prescrição de concentrado de plaquetas foi maior em crianças com hemorragia ativa. Houve propensão à solicitação de volumes excessivos e de subtipos de plaquetas sem justificativa pelos protocolos transfusionais atuais. Esses dados reforçam a necessidade de o ensino da medicina transfusional ser mais valorizado na graduação e na residência médica e de o hospital, em conjunto com o Comitê Transfusional, realizar educação continuada sobre o tema quando identificar a necessidade.
